# Serum exosomes miR-206 and miR-549a-3p as potential biomarkers of traumatic brain injury

**DOI:** 10.1038/s41598-024-60827-8

**Published:** 2024-05-02

**Authors:** Yajun Yang, Yi Wang, Panpan Li, Feirong Bai, Cai Liu, Xintao Huang

**Affiliations:** 1 Department of Neurosurgery, The First Hospital of Shanxi Medical University, Taiyuan, China; 2 The First School of Clinical Medicine, Shanxi Medical University, Taiyuan, China; 3 Department of Neurosurgery, Luxian People’s Hospital, Luzhou, China

**Keywords:** TBI, Biomarker, Exosomes, miR-206, miR-549a-3p, Molecular biology, Neuroscience, Neurology

## Abstract

Traumatic brain injury (TBI) is one of the leading causes of death and disability worldwide. However, effective diagnostic, therapeutic and prognostic biomarkers are still lacking. Our research group previously revealed through high-throughput sequencing that the serum exosomes miR-133a-3p, miR-206, and miR-549a-3p differ significantly in severe TBI (sTBI), mild or moderate TBI (mTBI), and control groups. However, convincing experimental evidence is lacking. To solve this problem, we used qPCR in this study to further verify the expression levels of serum exosomes miR-133a-3p, miR-206 and miR-549a-3p in TBI patients. The results showed that the serum exosomes miR-206 and miR-549a-3p showed good predictive value as biomarkers of TBI. In addition, in order to further verify whether serum exosomes miR-206 and miR-549a-3p can be used as potential biomarkers in patients with TBI and to understand the mechanism of their possible effects, we further determined the contents of SOD, BDNF, VEGF, VEGI, NSE and S100β in the serum of TBI patients. The results showed that, serum exosomes miR-206 and miR-549a-3p showed good correlation with BDNF, NSE and S100β. In conclusion, serum exosomes miR-206 and miR-549a-3p have the potential to serve as potential biomarkers in patients with TBI.

## Introduction

Traumatic brain injury (TBI) is a global public health problem with extremely high mortality and disability rates, especially in low—and middle-income countries^1–3^. In recent years, with the advancement of commonly used clinical diagnostic and treatment tools, including clinical indicators (Glasgow Coma Scale score), imaging examinations that include computed tomography and magnetic resonance imaging, and intracranial pressure monitoring, the diagnosis, treatment planning, and prognosis of TBI have improved. However, these methods cannot dynamically and quickly analyze a patient's condition in real-time in the clinical environment. Thus, there has been no significant reduction in the disability and mortality rates of patients^4^. Therefore, it is imperative to identify a reliable biomarker to assess the severity of TBI, predict recovery, and measure treatment efficacy in clinical practice. In TBI, fluid biomarkers have the potential to be used for the rapid diagnosis and accurate prediction of prognosis. Several potential biomarkers have been identified in cerebrospinal fluid (CSF) and blood^5,6^. The protein biomarkers brain-derived neurotrophic factor (BDNF), S100 calcium-binding protein β (S100B), and neuron-specific enolase (NSE) have been studied as blood biomarkers for TBI, but are limited by the low concentration of proteins in the blood and the inability to cross the blood–brain barrier (BBB)^7^. CSF sampling has limited applicability in mild TBI cases. ;However, mild cases account for 75% of emergency cases, and invasive procedures tend to increase the risk of infection in patients with low clinical utility^8^.

With the development of high-throughput sequencing technologies, new biomarker exosomes have gradually become recognized. Exosomes are widely present in various body fluids, such as blood, CSF, and saliva. Exosomes carry and transport microRNAs (miRNAs), messenger RNA (mRNA), lipids, proteins, and other signaling molecules; regulate the physiological state of cells and course of diseases; and are important diagnostic and therapeutic tools in clinical applications. miRNAs are a class of small noncoding RNAs involved in the post-transcriptional regulation of molecular functions, including mRNA degradation and mediation of protein synthesis^9,10^. miRNAs are also involved in the control of multiple biological processes, including cellular metabolism and regulation, with a single miRNA capable of regulating hundreds of target mRNAs^11,12^. Based on these properties, miRNAs have emerged as candidate biomarkers for the diagnostic, prognostic, and therapeutic effects of TBI^13,14^. Compared with traditional protein biomarkers, miRNAs may have higher sensitivity for the diagnosis of TBI because of their exceptional stability in peripheral blood, pronounced ability to cross the BBB, and protection of exosomes^15^. Previous studies have identified miRNAs as potential biomarkers of blood-based TBI^10,12,16^.

We have previously used high-throughput sequencing methods to show statistical differences in the expression of serum exosomes miR-133a-3p, miR-206, and miR-549a-3p in severe TBI (sTBI) and mild or moderate TBI (mTBI) patients compared to controls^17^. In this study, qPCR was further used to verify whether there were statistical differences in the expression of serum exosomes miR-133a-3p, miR-206 and miR-549a-3p between sTBI, mTBI and the control group, and to further explore the possible mechanism of their effects.

## Materials and methods

### Sample collection

The samples were collected from 01 January 2021 to 01 December 2022 from 15 sTBI patients (mean age 48.10 ± 9.60 years) and 15 mTBI patients (mean age 52.9 ± 11.2 years) admitted to the Department of Neurosurgery and Emergency Medicine of the First Affiliated Hospital of Shanxi Medical University. All included patients were confirmed by two trained neurosurgeons. The control group comprised 15 patients who underwent routine health examinations at the First Clinical Medical College of Shanxi Medical University. Coma was assessed using the most recent GCS criteria. Severity was assessed using total speech, movement, and eye opening scores as follows: mild or medium TBI: 9 points ≤ GCS ≤ 15 points; severe TBI: 3 points ≤ GCS ≤ 8 points.. Based on the GCS scores, 15 patients with mild-to-medium TBI and 15 patients with severe TBI were included. Inclusion Criteria were age between 18 and 65 years, admission within 24 h of onset, and no surgical intervention. Exclusion criteria were open head injury, major injuries to other organs and long bone fractures, and other chronic diseases. Through oral medical history taking and querying of our hospital's medical record system, various clinical data of patients were collected. Specific items were age, gender, history of hypertension, diabetes history, smoking history, drinking history, causes of trauma, and others. All were accurately recorded. Six milliliters of peripheral blood from each subject was collected in EDTA-treated tubes. Two centrifugation protocols (1,800 g for 30 min and 13,000 g for 2 min) were performed to transfer the obtained serum to a 200 μL to 1.5 mL, ultra-low temperature resistant (− 192 °C), threaded mouth cryopreservation tube with at least ≥ 0.1 mL per sample. Each sample was snap-frozen in liquid nitrogen for 1 h and stored at − 80 °C for later use. This study was reviewed and approved by the Ethics Committee of Shanxi Medical University(K-K0100) on April 26, 2022 (see in Supplement S2). In addition, in accordance with national legislation and institutional requirements, we obtained written informed consent from patients or legal guardians/close relatives participating in this study.

### Extraction of serum exosomes

Serum samples were used for exosome isolation with Umibio® exosome isolation kits (Umibio, Cat. No: UR52136, China) according to the manufacturer's instructions.In brief, an initial spin was performed at 3000×*g* 4 °C for 10 min and 10,000×*g* 4 °C for 20 min for each sample to remove cells and debris, then the corresponding amounts of reagents were added proportional to the starting sample volume, according to the manufacturer's instructions. Mixtures were vortexed and incubated at 4 °C for up to 2 h and then centrifuged at 10,000×*g* 4 °C for 60 min to precipitate exosome pellets. Pellets were resuspensed with 1 × PBS and purified with Exosome Purification Filter at 3000×*g* 4 °C for 10 min. The resuspension volume for exosome pellets was 200 μL for 20 mL starting volumes according to the manufacturer's instructions. All exosomes were stored at − 80 °C immediately after isolation until further analysis.

### Transmission electron microscopy (TEM)

The morphological characteristics of exosomes were visualized using transmission electron microscopy (TEM; JEOL-1230; Tokyo, Japan). Briefly, 30 µL of exosome samples were placed on a sheet of parafilm, and a 100-mesh copper grid was transferred to drops of exosomes with forceps for 10 min. Phosphotungstic acid was then used to stain the grid for 15 s and dried at 23 °C and 30 °C.

### Nanoparticle tracking analyzer (NTA)

The size of exosomes was detected by nanoparticle tracking analysis (NTA) using ZetaView PMX110 (Particle Metrix, Meerbusch, Germany) and its accompanying software. Isolated exosome samples were appropriately diluted using 1X PBS buffer (Biological Industries, Israel) to measure particle size and concentration.

### Western blotting (WB)

In order to determine whether the extracted serum exosomes had the characteristic proteins of exosomes, Western blot analysis was used to detect the exosome surface marker (TSG101, anti-CD63) and GAPDH as the internal reference protein. The two proteins mentioned above need to be positive to meet the requirements of exosome characteristic proteins. After determination of BCA protein concentration (Beyotime, Shanghai, China), SDS-PAGE was used to isolate proteins using the following gel: TSG101 and CD-63 (loaded 10 µg per strip) were separated by 10% SDS-PAGE and 5% concentrated gel. Protein transfer to polyvinylidene fluoride (PVDF) membrane (Immobilon-P, USA) is performed under standard wet transfer conditions. The PVDF membrane was blocked in TBST solution containing 10% skimmed milk at room temperature for 1 h. After four washes with PBST (8 min each), the PVDF membrane incubates the following primary antibodies overnight at 4 °C: In order to determine whether the extracted serum exosomes had the characteristic proteins of exosomes, Western blot analysis was used to detect the exosome surface marker (TSG101, anti-CD63) and GAPDH as the internal reference protein. The two proteins mentioned above need to be positive to meet the requirements of exosome characteristic proteins. After determination of BCA protein concentration (Beyotime, Shanghai, China), SDS-PAGE was used to isolate proteins using the following gel: TSG101 and CD-63 (loaded 10 µg per strip) were separated by 10% SDS-PAGE and 5% concentrated gel. Protein transfer to polyvinylidene fluoride (PVDF) membrane (Immobilon-P, USA) is performed under standard wet transfer conditions. The PVDF membrane was blocked in TBST solution containing 10% skimmed milk at room temperature for 1 h. After four washes with PBST (8 min each), PVDF membranes were incubated overnight at 4 °C for the following primary antibodies: anti-CD63, anti-TSG101, and anti-GAPDH antibodies (all 1:1000 diluted). After four washes with PBST (8 min each), the PVDF membrane was incubated at room temperature with a secondary antibody (Abcam, Cambridge, USA, Cat. No. AB205719) bound to horseradish peroxidase (HRP) for 1 h. After four washes with PBST (8 min each), the film was developed using Immobilon Western HRP luminescence reagent (Millipore, USA) and the results were analyzed. And anti-GAPDH antibodies (both 1:1000 diluted). After four washes with PBST (8 min each), the PVDF membrane was incubated at room temperature with a secondary antibody (Abcam, Cambridge, USA, Cat. No. AB205719) bound to horseradish peroxidase (HRP) for 1 h. After four washes with PBST (8 min each), the film was developed using Immobilon Western HRP luminescence reagent (Millipore, USA) and the results were analyzed.

### Quantitative real-time PCR (qPCR)

To evaluate the levels of miR-133a-3p, miR-206 and miR-549a-3p in exosomes, total RNA was extracted from the vesicles using Trizol reagent (Invitrogen) and quantified using Nanodrop (Thermo Scientific, Waltham, USA). Stem-loop qPCR (Taq-Man) was employed to measure the expression levels of miRNA, with U6 serving as the reference gene. For miRNA level assessment, cDNA was used as the template for qPCR using TB GreenTM Premix Ex TaqTM II (Takara; RR820A) and GAPDH as the reference gene. The miRNA qPCR primer sets were obtained from RiboBio, and the miRNA qPCR primers were synthesized by Sangon (Shanghai, China). The comparative Ct method (2 − ΔΔCt) was used to calculate the values of each sample, with triplicate measurements performed for each sample. The sequence of primers used is as follows:
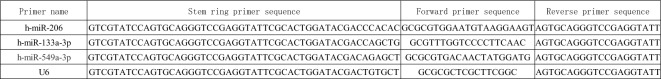


2.7. Determination of serum levels of superoxide dismutase (SOD), BDNF, vascular endothelial growth factor (VEGF), vascular endothelial growth inhibitor (VEGI), NSE, and S100β.

The levels of serum SOD, BDNF, VEGF, VEGI, NSE, and S100β were measured using enzyme-linked immunosorbent assay (ELISA) method, and the OD values of each sample were determined at a wavelength of 450 nm.

### Statistical analyses

Statistical analyses were performed using SPSS 27.0 software (IBM, Armonk, NY, USA), Experimental data were mapped using Graphpad Prism 7.0 software (Graphpad, San Diego, CA, USA). The measurement data that conformed to the normal distribution application is expressed as mean ± standard deviation. Data that did not conform to the normal distribution are expressed as median ± quartile range. When comparing data of metrological groups, normal distribution and homogeneity of variance tests were confirmed. At least one group did not conform to the normal distribution or homogeneity of variance test, and the Mann–Whitney U test or Kruskal–Wallis H-rank sum test was used for intergroup comparison. Counting data were described using the composition ratio, and the chi-square test was used for intergroup comparisons. Spearman’s correlation analysis was used for the correlation analysis; differences were considered statistically significant at P < 0.05. A receiver operating characteristic (ROC) curve was established to evaluate the specificity, sensitivity, and best predictive value of the target miRNAs in exosomes for predicting sTBI and poor prognosis of sTBI. Statistical significance was set at P < 0.05.

### Ethics statement

According to the requirements of local legislation and institutions, this study passed the ethical review and approval of the Ethics Committee of the First Hospital of Shanxi Medical University. In addition, in accordance with national legislation and institutional requirements, we obtained written informed consent from patients or legal guardians/close relatives participating in this study.

## Results

### Clinical data

A total of 45 participants were included in this study, comprising 15 in the control group and 30 in the TBI group (15 in the severe TBI and 15 in the mild and medium TBI groups). The basic clinical data of the 45 enrolled subjects, including age, sex, smoking history, drinking history, and cause of injury, were collected, and no statistical differences were found after statistical analysis (P > 0.05). Statistical descriptions and comparisons of relevant clinical data are presented in Table 1.

**Table 1:** Comparison of basic clinical data of each group.

Parameter	Control (n = 15)	mTBI (n = 15)	sTBI (n = 15)	F/X^2^	*P* value
Age (years)	51.47 ± 10.24	52.9 ± 11.2	48.10 ± 9.60	0.844	0.437
Male [example (%)]	13 (86.7)	12 (80.0)	14 (93.3)	1.154	0.562
History of long-term smoking [example (%)]	3 (20.0)	6 (40)	1 (6.7)	4.886	0.087
History of long-term alcohol consumption [example (%)]	5 (33.7)	6 (40)	1 (6.7)	4.773	0.092
Cause of injury				5.536	1.137
Traffic accident injuries [example (%)]	–	7 (46.7)	8 (53.3)		
Fall injuries [example (%)]	–	3 (20.0)	1 (6.7)		
Fall injuries from height [example (%)]	–	1 (6.7)	5 (33.3)		
Other [Example (%)]	–	4 (26.7)	1 (6.7)		
History of hypertension	1 (13.3)	2 (13.3)	3 (20.0)	1.154	0.562
History of diabetes	2 (6.7)	1 (6.7)	1 (6.7)	0.549	0.760

### TEM of morphology of exosomes

**Figure 1:**
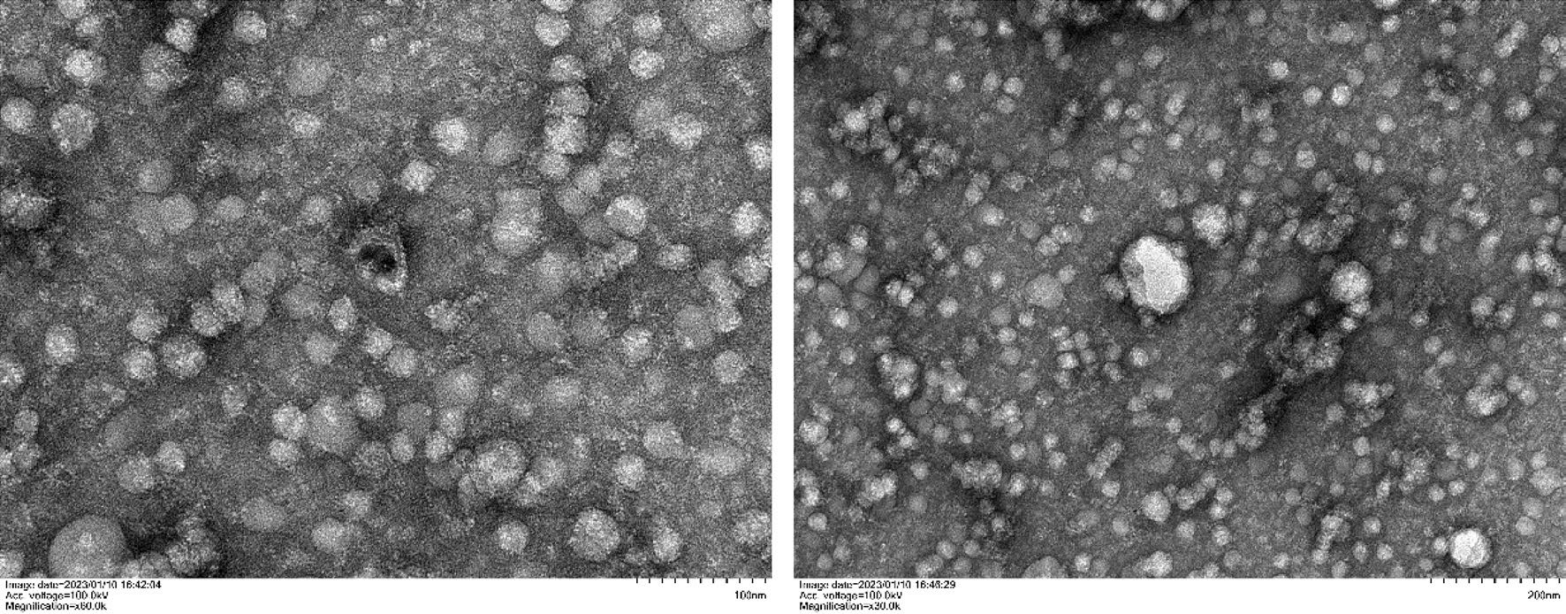
Morphological characterization of exosomes isolated from serum samples by transmission electron microscopy. Bar, 100 nm and 200 nm.

Exosomes observed by TEM appeared as circular bilayer membrane vesicles with diameter of 30–150 nm (see Fig. 1), consistent with previous literature data^18^.

### Concentration particle size distribution of exosomes

**Figure 2:**
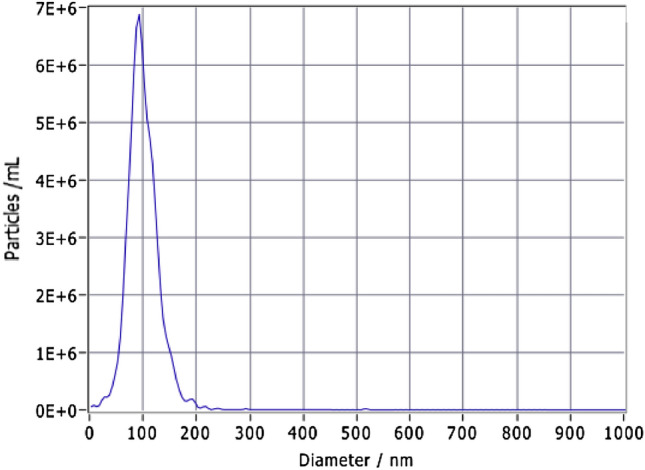
Concentration and size of exosomes were analyzed by the nanoparticle tracking analysis.

NTA showed that the particle concentration of exosomes was between 10^7^ ~ 10^13^ particles/mL and the diameter distribution was between 30 and 200 nm (Fig. 2). These findings are consistent with previously reported data^19^.

### Identification of surface marker proteins of exosomes

**Figure 3:**
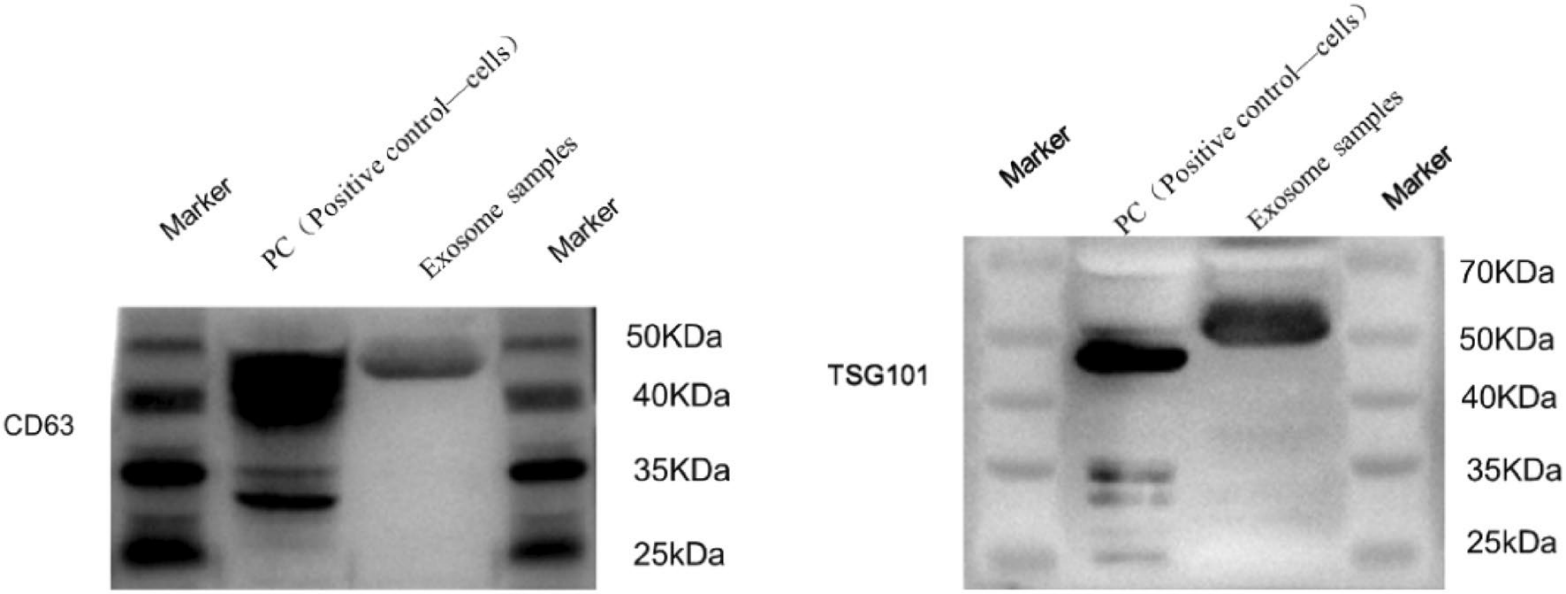
Expression of exosomal protein markers (CD63 and TSG101) in exosomes isolated from serum samples was assessed by Western blot analysis.

Expression of CD63 and TSG101 proteins in exosomes was detected by western blotting. The bands of proteins extracted from EXO had the same molecular weight as the positive control bands, confirming the expression of CD63 and TSG101 (Fig. 3).The original exposure image can be found in Supplement S1. These findings are consistent with previous literature data^20^.

### Comparison of serum exosomal miR-133a-3p, miR-206 and miR-549a-3p expression levels in sTBI, mTBI, and control groups

**Figure 4:**
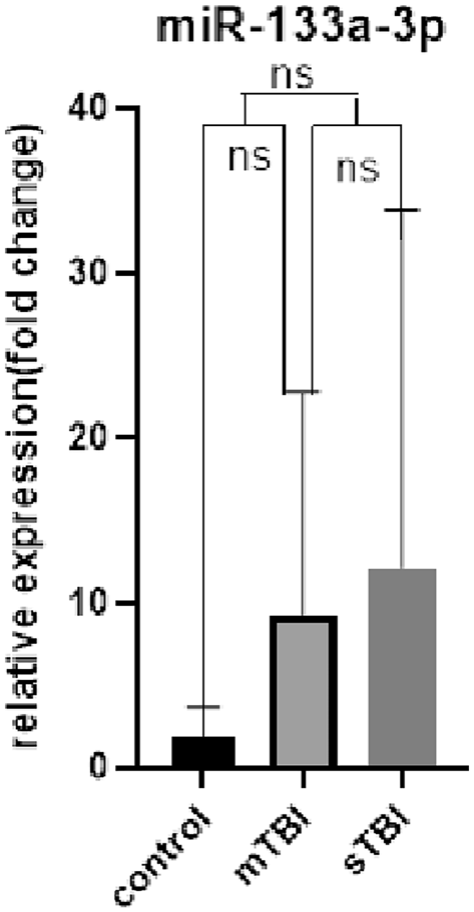
ns: sTBI, mTBI and control were compared with pairs, *P* > 0.05.

**Figure 5:**
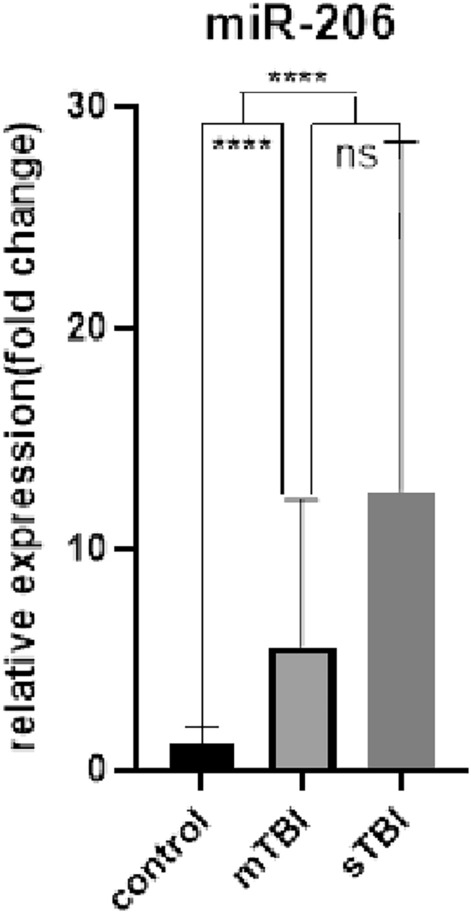
ns: sTBI versus mTBI, P > 0.05; ****: sTBI, mTBI versus control, *P* < 0.0001.

**Figure 6:**
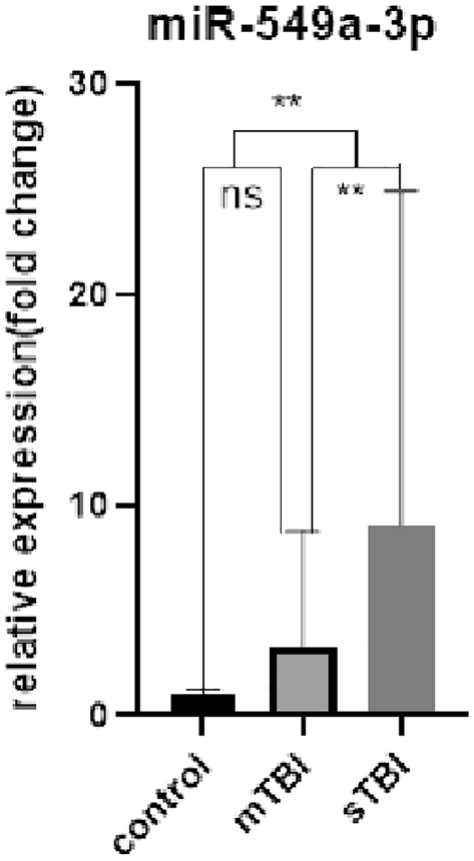
ns: mTBI compared with control, *P* > 0.05; **: mTBI and control compared with sTBI, P > 0.01.

The serum exosomal miRNA levels of the sTBI, mTBI, and control groups were determined. The results (Table 2, Figs. 4, 5, 6) indicated no significant difference in serum expression of exosomal miR-133a-3p (P > 0.05). The expression level of serum exosomal miR-206 showed an upward trend in the sTBI and mTBI groups compared with the control group (P < 0.01), with no significant statistical difference between the sTBI and mTBI groups (P > 0.05). The expression level of serum exosomal miR-549a-3p was greater in the sTBI group compared with the control group (P < 0.01), with no significant statistical difference between the mTBI and control groups (P > 0.05). The sTBI group showed a significant upward adjustment trend of miR-549a-3p compared with the mTBI group (P < 0.01).

**Table 2:** Expression levels of serum exosomal miR-133a-3p, miR-206, and miR-549a-3p in sTBI, mTBI patients and controls.

	sTBI (n = 15)	mTBI (n = 15)	Control (n = 15)
miR-133a-3p(fold change)	0.985 (0.462, 18.860)	2.522 (0.330, 16.281)	0.941 (0.357, 3.938)
miR-206(fold change)	5.276 (2.696, 19.948)	3.067 (1.902, 7.118)	1.068 (0.513, 1.685)
miR-549a-3p(fold change)	4.843 (1.089, 7.791)	0.872 (0.534, 3.105)	1.059 (0.958, 1.130)

### Comparison of SOD, BDNF, VEGF, VEGI, NSE, S100β expression levels in sTBI, mTBI, and control groups

Statistical differences in the expressions of SOD, BDNF, VEGI, NSE, and S100β were eident between the three groups (P < 0.01). There was no significant statistical difference in the expression level of VEGF between the three groups (P > 0.05) (Table 3).

**Table 3:** Comparison of SOD, BDNF, VEGF, VEGI, NSE and S100β expression levels in the sTBI, mTBI, and control groups.

	Control (n = 15)	mTBI (n = 15)	sTBI (n = 15)	F	*P* value
SOD (ng/mL)	252.66 ± 38.01	82.09 ± 84.56	190.66 ± 33.15	19.984	< 0.01
BDNF (ng/mL)	12.49 ± 2.21	7.42 ± 1.82	9.64 ± 2.54	24.599	< 0.01
VEGF (pg/mL)	111.35 ± 24.97	120.65 ± 31.92	108.68 ± 27.25	1.602	0.208
VEGI (pg/mL)	69.68 ± 14.77	123.98 ± 27.24	110.03 ± 19.51	22.156	< 0.01
NSE (ng/mL)	15.43 ± 5.88	30.85 ± 4.42	28.5922 ± 6.15	33.403	< 0.01
S100β (ng/mL)	4.02 ± 1.03	6.43 ± 0.90	6.11 ± 1.23	20.957	< 0.01

### Correlation analysis of serum exosomal miR-206 expression levels with SOD, BDNF, VEGI, NSE, and S100β in TBI patients

The correlation between the expression level of serum exosomal miR-206 in the TBI group and the contents of SOD, BDNF, VEGI, NSE and S100β in serum was analyzed. The r values were 0.180, 0.329, 0.043, − 0.426, and − 0.235, respectively. The specific values were shown in Table 4. The results showed that the expression of serum exosome miR-206 was negatively correlated with the expression of S100β and NSE (P < 0.05), positively correlated with the expression of BDNF (P < 0.01), and had no statistical difference with the expression of SOD and VEGI (P > 0.05).

**Table 4:** Correlation between serum exosomal miR-206 expression levels and SOD, BDNF, VEGI, NSE, and S100β in TBI patients.

Variable	r	*P* value
SOD	0.180	0.126
BDNF	0.329	0.004
VEGI	0.043	0.714
NSE	− 0.426	< 0.01
S100**β**	− 0.235	0.044

### Correlation analysis of serum exosome miR-549a-3p expression levels with SOD, BDNF, VEGI, NSE, and S100β in the sTBI group

The expression level of serum exosomal miR-549a-3p in the sTBI group was analyzed in relation to the content of SOD, BDNF, VEGI, NSE, and S100β. The r values were 0.166, 0.458, − 0.281, − 0.589, and − 0.610, respectively. The specific values are shown in Table 5. The results showed that the expression of serum exosome miR-549a-3p was negatively correlated with the expression of S100β and NSE (P < 0.01), positively correlated with the expression of BDNF (P < 0.01), and had no statistical difference with the expression of SOD and VEGI (P > 0.05).

**Table 5:** Correlation between serum exosomal miR-549a-3p expression levels and SOD, BDNF, VEGI, NSE, and S100β in the sTBI group.

Variable	r	*P* value
SOD	0.166	0.349
BDNF	0.458	0.006
VEGI	− 0.281	0.107
NSE	− 0.589	< 0.01
S100**β**	− 0.610	< 0.01

### Predictive value of serum exosomal miR-206 in patients with TBI

**Figure 7:**
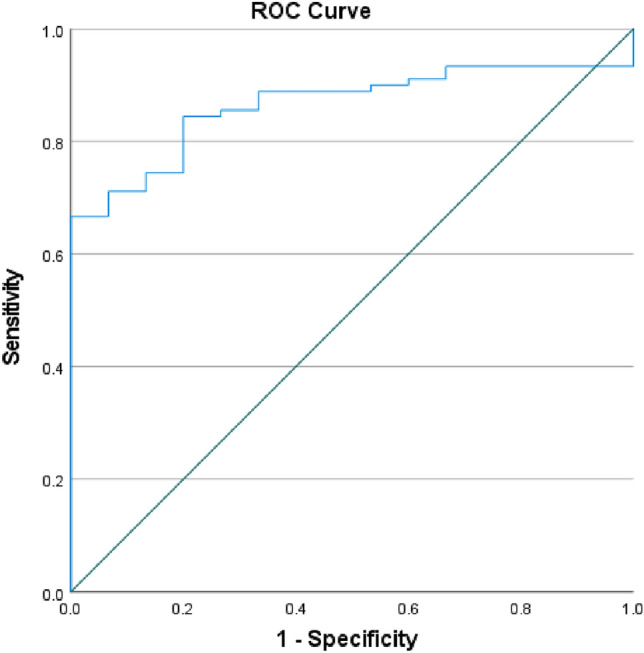
Predictive value of serum exosomal miR-206 for TBI.

The expression of serum exosomal miR-206 in TBI patients increased. Thus, we analyzed the ROC curve of TBI diagnostic ability based on the relative expression level of serum exosome miR-206 to evaluate the diagnostic value and application potential of serum exosomal miR-206 for TBI. Serum exosomal miR-206 was used to diagnose sTBI based on an area under the ROC curve (AUC) value of 0.86 (95% CI 0.79–0.94; P < 0.01), optimal cut-off value of 2.65, sensitivity of 67%, and specificity of 93% (Fig. 7).

### Predictive value of serum exosomal miR-206 and miR-549a-3p in patients with sTBI

**Figure 8:**
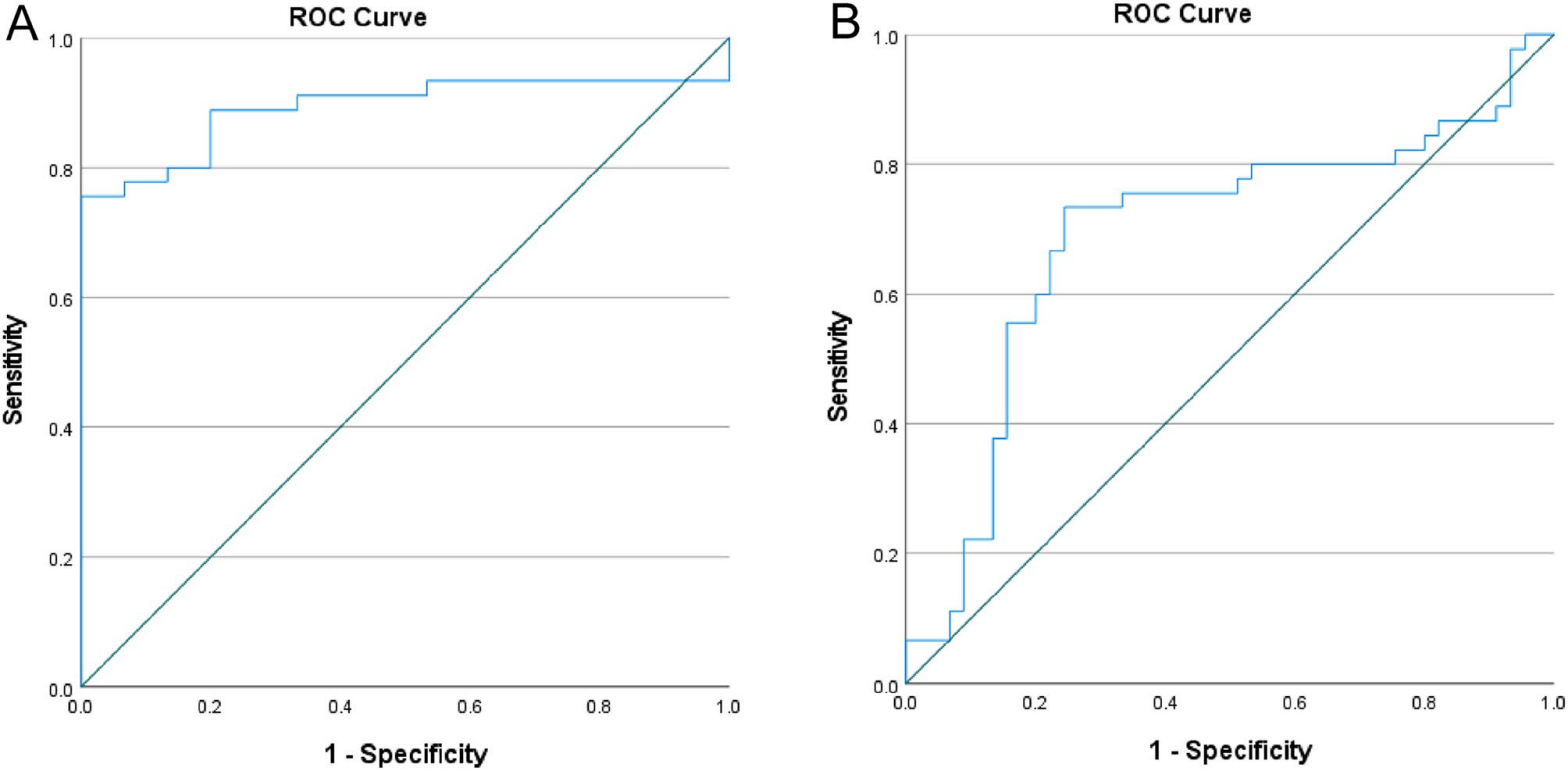
(**A**) Predictive value of serum exosome miR-206 for sTBI, (**B**) Predictive value of serum exosomal miR-549a-3p for sTBI.

The expression of serum exosomal miR-206 and miR-549a-3p in sTBI patients increased. Thus, we analyzed the ROC curve of sTBI on the basis of the relative expression level of serum exosomal miR-206 and miR-549a-3p,Serum exosomal miR-206 could be used to diagnose sTBI, based on the AUC of 0.89 (95% CI 0.81–0.98; P < 0.01), optimal cut-off value of 2.65, sensitivity of 76%, and specificity of 93% (see Fig. 8A).Serum exosomal miR-549a-3p could diagnose sTBI based on the AUC of 0.69 (95% CI 0.58–0.81; P < 0.01), optimal cut-off value of 2.81, sensitivity of 73%, and specificity of 76% (see Fig. 8B).

### Predictive value of serum exosomal miR-206 and miR-549a-3p in sTBI patients with poor prognosis

**Figure 9:**
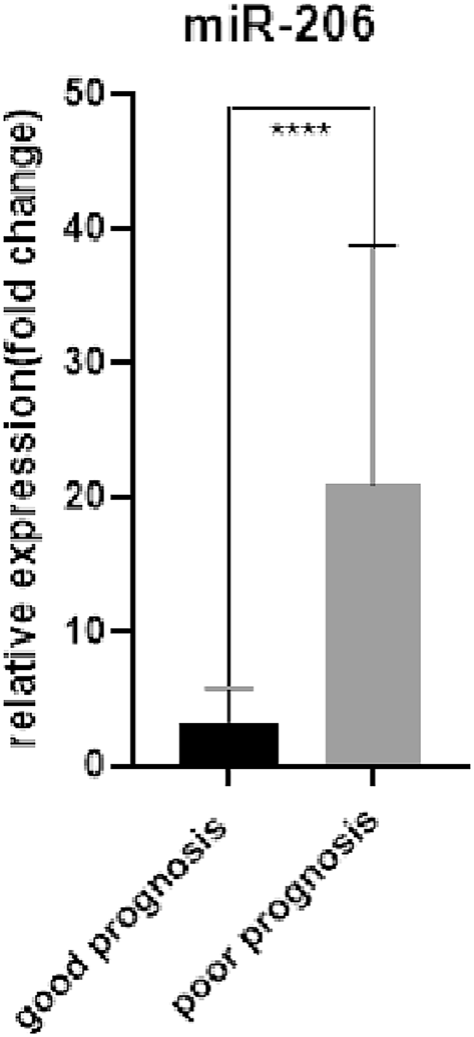
****: Good prognosis group versus poor prognosis group, P < 0.0001.

**Figure 10:**
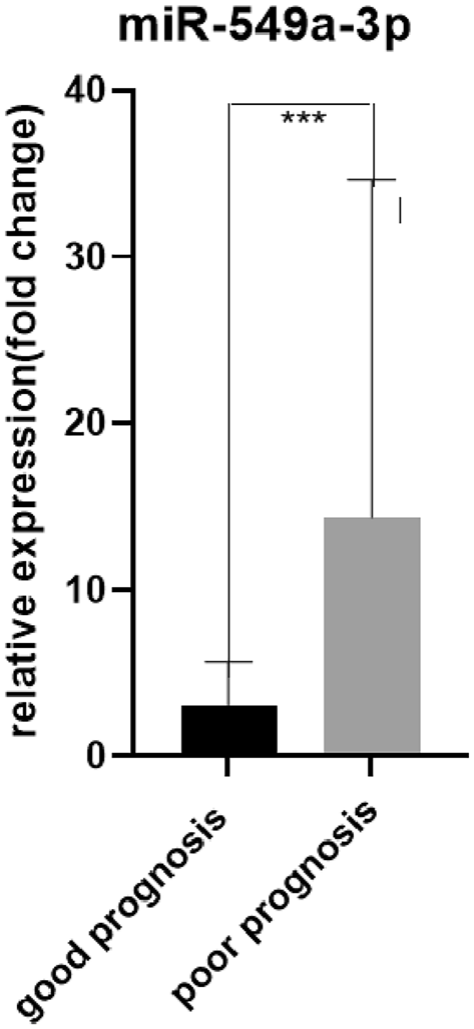
***: Good prognosis group versus poor prognosis group, P < 0.0001.

**Figure 11:**
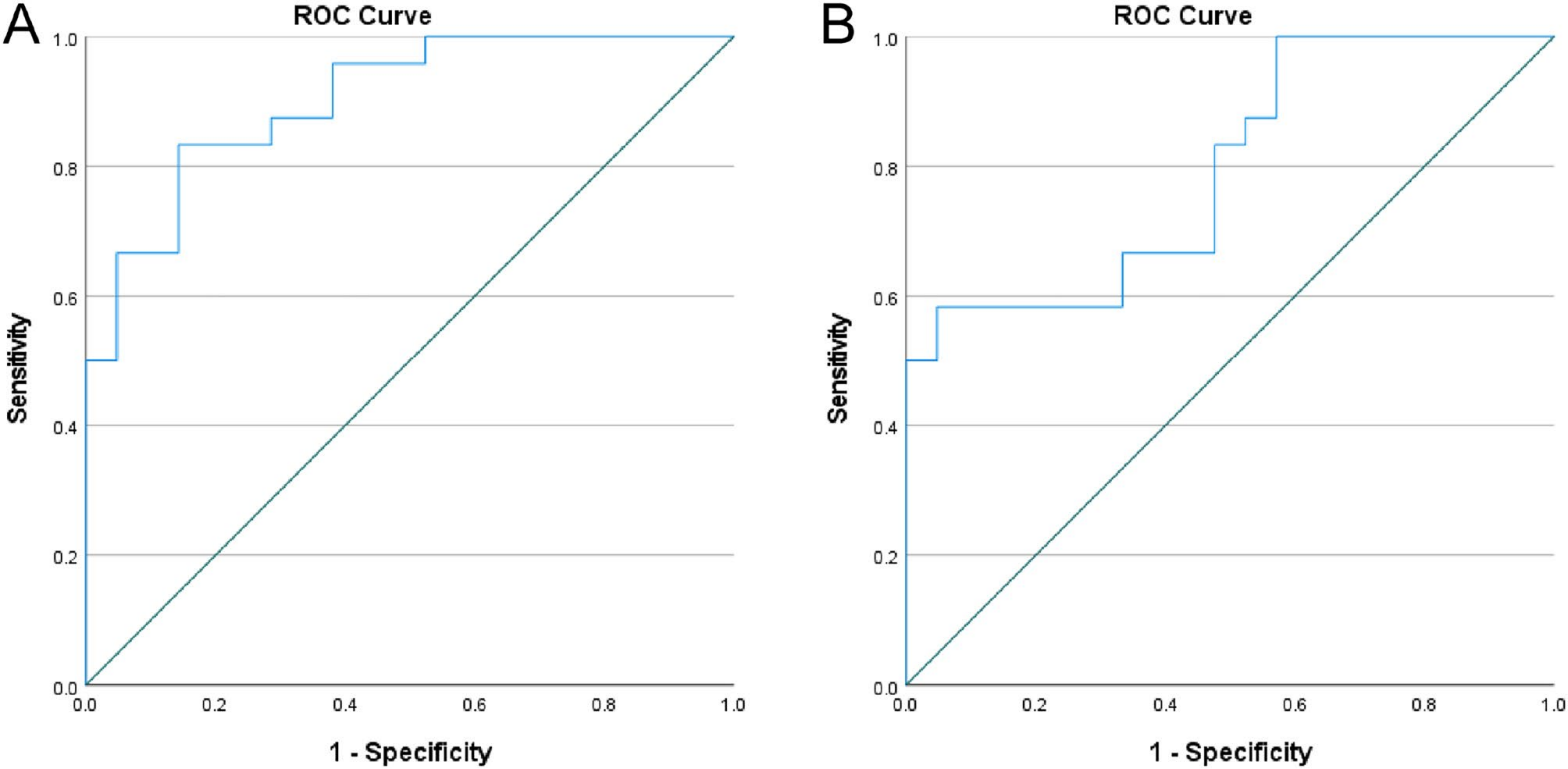
(**A**) Predictive value of serum exosome miR-206 for poor sTBI prognosis, (**B**) Predictive value of serum exosomal miR-549a-3p for poor sTBI prognosis.

In order to further study the predictive value of serum exosomal miR-206 and miR-549a-3p in sTBI patients with poor prognosis, we divided sTBI patients into a good prognosis group and poor prognosis group according to GOS score, among which the good prognosis group: 4 points ≤ G0S ≤ 5 points, and the poor prognosis group:1 point ≤ GOS ≤ 3 points. A comparison of the data between the good and poor prognosis groups is shown in Table 6. First, we analyzed the expression levels of serum exosomal miR-206 and miR-549a-3p in the good and poor prognosis groups and found that the expression levels of serum exosomal miR-206 and miR-549a-3p in those with poor prognosis were significantly higher than for those with good prognosis (P < 0.01; Figs. 9 and 10). ROC curve analysis was performed to evaluate the diagnostic value and application potential of serum exosomal miR-206 and miR-549a-3p in STBI patients with poor prognosis. Serum exosomal miR-206 could diagnose sTBI based on an AUC of 0.90 (95% CI 0.82–0.99; P < 0.01), optimal cut-off value of 5.27, sensitivity of 83%, and specificity of 86% (see Fig. 11A). Serum exosomal miR-549a-3p could also diagnose sTBI based on an AUC of 0.80 (95% CI 0.67–0.93; P < 0.01), optimal cut-off value of 6.56, sensitivity of 58%, and specificity of 95% (see Fig. 11B).

**Table 6:** Comparison of clinical data between sTBI patients with poor and good prognosis.

Project	Poor prognosis (n = 8)	Good prognosis (n = 7)	t/X^2^/Z	*P* value
Age (years)	44.75 ± 9.41	52.00 ± 9.00	− 1.519	0.153
Male [example (%)]	8 (100.0)	6 (85.7)	1.224	0.268
History of long-term smoking [example (%)]	4 (50.0)	2 (28.6)	0.714	0.398
History of long-term alcohol consumption [example (%)]	0 (0.0)	1 (14.3)	1.224	0.268
History of hypertension	2 (25.0)	1 (14.3)	0.268	0.605
History of diabetes	1 (12.5)	0 (0.0)	0.938	0.333
miR-206	13.461 (5.676, 37.253)	2.800 (1.544, 4.619)	− 4.618	< 0.01
miR-549a-3p	7.475 (3.624, 14.390)	3.359 (0.187, 5.492)	− 3.390	< 0.01

## Discussion

TBI has a high incidence and mortality worldwide, especially in low- and middle-income countries^21,22^. However, currently there is still a lack of effective strategies for diagnosis, treatment, and prognosis evaluation^23,24^. So there is an urgent need to develop new biomarkers. Recent biological studies have identified several novel biomarkers (e.g., miRNAs), EXO, and exosomal miRNAs), which have shown strong advantages in the diagnosis, treatment, and prognosis of diseases, and have therefore been widely studied in various diseases, including TBI^22^. In this study, we further verified the expression of serum exosomes miR-133a-3p, miR-206 and miR-549a-3p in TBI patients by using qPCR assay, and the results showed partial consistency with the previous results^17^. This study provides preliminary evidence for the potential of serum exosomes miR-206 and miR-549a-3p as novel biomarkers of TBI. However, our results showed that the expression of serum exosome miR-549a-3p was more statistically significant in patients with sTBI. In addition, we demonstrated statistically that serum exosomes miR-206 and miR-549a-3p, as biological markers, showed good predictive value in TBI or sTBI.

miR-206 is a member of the miR-1 family and has been shown to be involved in the pathogenesis of various malignant and non-malignant diseases^25,26^. Multiple studies have demonstrated that miR-206 participates in processes such as cell apoptosis and regulation of endothelial cell proliferation, and it can serve as a biomarker for various neurological disorders^27,28^. miR-549a-3p is a microRNA, which is the mature product of the miR-549 gene. miR-549a-3p has been found to play important regulatory roles in various biological processes^29^. Studies have shown that miR-549a-3p plays a crucial role in the occurrence and development of tumors^29^. It can affect biological processes such as cell proliferation, apoptosis, invasion, and metastasis by regulating the expression of multiple target genes^29^. In addition, miR-549a-3p is also associated with the occurrence and development of various diseases, including cardiovascular diseases, neurological disorders, and immune system diseases^29^. Unfortunately, there have been no reports on the role of serum exosomes miR-206 and miR-549a-3p in traumatic brain injury. Our study fills this gap.

In order to further study the mechanism of action of serum exosomes miR-206 and miR-549a-3p, we further determined the contents of SOD, BDNF,VEGF, VEGI, NSE, and S100β in peripheral blood serum of patients. The results showed that, in addition to VEGF, The other substances showed statistical difference in TBI patients. Further correlation analysis showed that serum exosomes miR-206 and miR-549a-3p showed good correlation with BDNF, NSE and S100β. NSE exists in the form of homologous dimers in mature neurons and neuroendocrine cells^30^. The biomarker S100 calc-binding protein β (S100β) is a protein located in glial cells of the central and peripheral nervous systems^31^. A number of studies have shown that the levels of NSE and S100β in sTBI patients are significantly increased^32–35^, which can promote neuroinflammation and have neurotoxic effects^30,36,37^, and can be used as biomarkers to judge the severity and prognosis of traumatic brain injury^30,34,38^. BDNF is one of the most widely distributed and widely studied neurotrophic factors in the mammalian brain, and is considered to be a guiding medium of functional and structural plasticity of the central nervous system (CNS), providing the function of an effective factor to protect against neurodegeneration^39^. A number of studies have shown that BDNF plays an important protective role in TBI and can improve the dysfunction caused by TBI^40,41^. Recent studies have shown that BMSCS-derived exosomes mediated by BDNF have a protective effect on TBI through miR-216a-5p, and HucmSCs-derived exosomes stimulated by BDNF promote neural network remodeling after traumatic brain injury^42,43^. Although our study found an association between serum exosomes miR-206 and miR-549a-3p and NSE, S100β and BDNF, further in vitro and in vivo experiments are needed to confirm their association and downstream signaling molecular mechanisms.

In conclusion, our study suggests that the serum exosomes miR-206 and miR-549a-3p may serve as potential targets for future TBI diagnosis and therapy. However, to truly determine their role in TBI, further in-depth studies based on cell models or animal models are needed. And the results of this study need further validation on cohorts with a larger sample size. In addition, the study had a limited number of observed cases, a wide age range, a sample from a single hospital, a lack of multi-center data and objective measures to determine TBI severity, and only GCS scores were available. Therefore, in future studies, we will expand the sample size, control the age of the sample, and supplement our study by including multi-center data and imaging data.

## Conclusions

In conclusion, our study suggests that serum exosomal miR-206 and miR-549a-3p have the potential to serve as novel biomarkers for TBI. Further research is needed to uncover the intricate regulatory networks in which they are involved in the pathogenesis of TBI.

### Supplementary Information

Supplementary Information 1.

Supplementary Information 2.

## Data Availability

The original contributions presented in this study are included in the article/supplementary material. Inquiries can be directed at the corresponding author.
