# Author Correction: Serum exosomes miR-206 and miR-549a-3p as potential biomarkers of traumatic brain injury

**DOI:** 10.1038/s41598-024-64962-0

**Published:** 2024-06-18

**Authors:** Yajun Yang, Yi Wang, Panpan Li, Feirong Bai, Cai Liu, Xintao Huang

**Affiliations:** 1 Department of Neurosurgery, The First Hospital of Shanxi Medical University, Taiyuan, China; 2 The First School of Clinical Medicine, Shanxi Medical University, Taiyuan, China; 3 Department of Neurosurgery, Luxian People’s Hospital, Luzhou, China

Correction to: *Scientific Reports* 10.1038/s41598-024-60827-8, published online 02 May 2024

The original version of this Article contained an error in the Funding section.

“This work was funded by the Research Project Supported by the Shanxi Scholarship Council of China (2020-169), the Fund Program for the Scientific Activities of Selected Returned Overseas Professionals in Shanxi Province (20210023), the Fundamental Research Program of Shanxi Province (202203021211017), and Mechanism of Human Umbilical Cord Blood Mesenchymal Stem Cells in Promoting the Repair of Peripheral Facial Paralysis in Rats (YZKJ2311004).”

now reads:

“This work was funded by the Research Project Supported by the Shanxi Scholarship Council of China (2020-169), the Fund Program for the Scientific Activities of Selected Returned Overseas Professionals in Shanxi Province (20210023), the Fundamental Research Program of Shanxi Province (202203021211017), and Science and Technology Project of Yingze District in Taiyuan City (YZKJ2311004).”

The original Article has been corrected.

