# Supplementary figures and images for: Serum exosomes miR-206 and miR-549a-3p as potential biomarkers of traumatic brain injury
Source: Sci Rep. 2024 May 2;14:10082. doi: 10.1038/s41598-024-60827-8 (PMC11066004; doi:10.1038/s41598-024-60827-8)

CD63-8.Tif

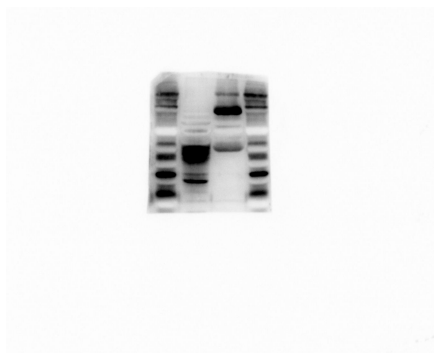

CD63-16.Tif

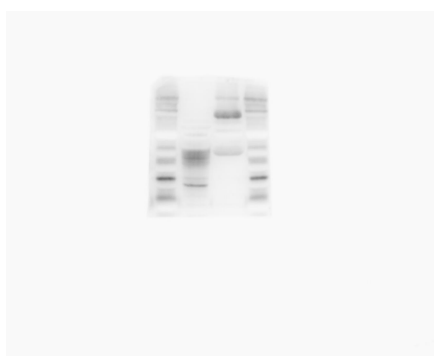

CD63-Merge.Tif

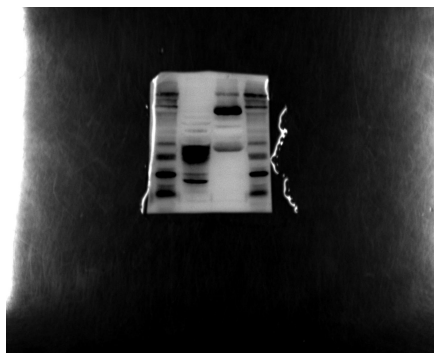

CD63-Merge.Tif-2.Tif

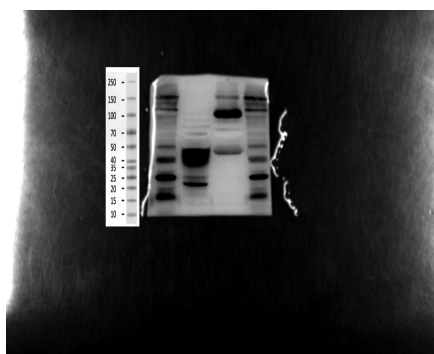

TSG101-8.Tif

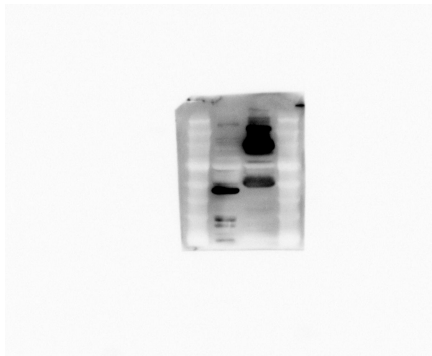

TSG101-16.Tif

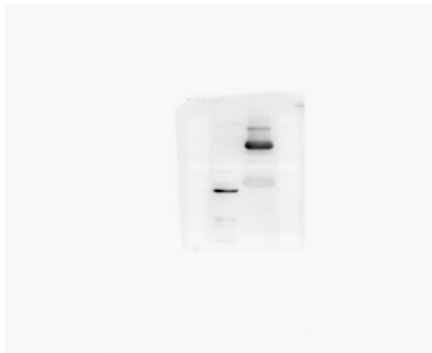

TSG101-Merge.Tif

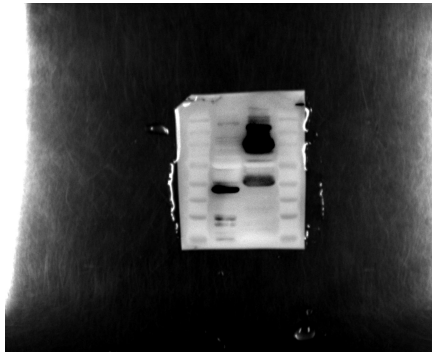

TSG101-Merge.Tif-2.Tif

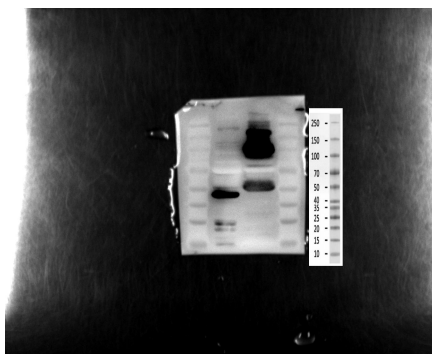

Supplement: Supplementary file 1 — Supplementary Information 1. [file 41598_2024_60827_MOESM1_ESM.pdf]

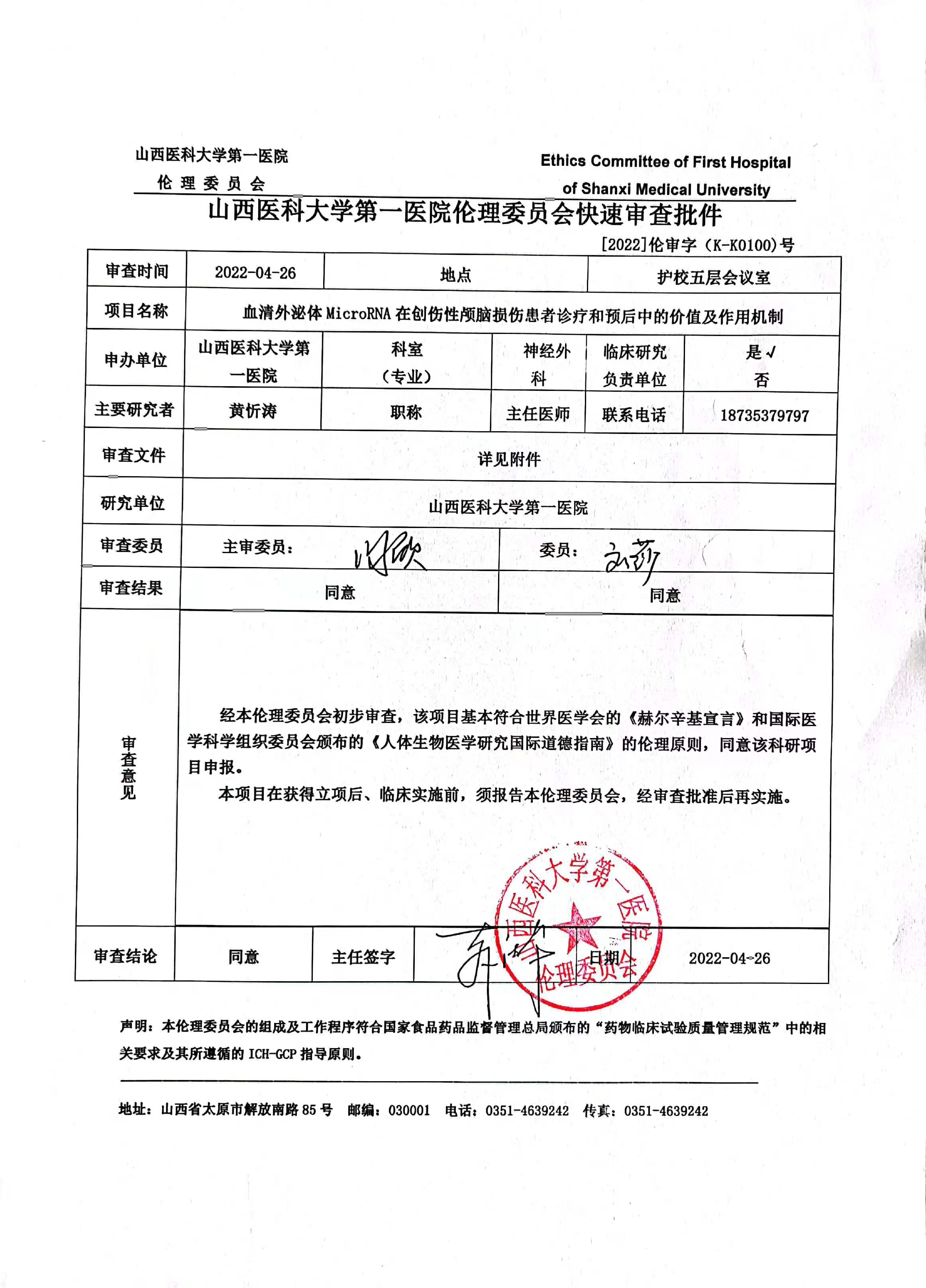

Supplement: Supplementary file 2 — Supplementary Information 2. [file 41598_2024_60827_MOESM2_ESM.jpg]
